# Frequentist p-values for large-scale-single step genome-wide association, with an application to birth weight in American Angus cattle

**DOI:** 10.1186/s12711-019-0469-3

**Published:** 2019-06-20

**Authors:** Ignacio Aguilar, Andres Legarra, Fernando Cardoso, Yutaka Masuda, Daniela Lourenco, Ignacy Misztal

**Affiliations:** 1 Instituto Nacional de Investigación Agropecuaria (INIA), 90200 Canelones, Uruguay; 2 UMR GenPhySE, INRA Toulouse, BP52626, 31326 Castanet Tolosan, France; 3 Department of Animal Science, Federal University of Pelotas, Rio Grande do Sul, Brazil; 4 Embrapa Pecuária Sul, Bagé, RS 96400-031 Brazil; 5 Department of Animal and Dairy Science, University of Georgia, Athens, GA USA

## Abstract

**Background:**

Single-step genomic best linear unbiased prediction (SSGBLUP) is a comprehensive method for genomic prediction. Point estimates of marker effects from SSGBLUP are often used for genome-wide association studies (GWAS) without a formal framework of hypothesis testing. Our objective was to implement p-values for single-marker GWAS studies within the single-step GWAS (SSGWAS) framework by deriving computational algorithms and procedures, and by applying these to a large beef cattle population.

**Methods:**

P-values were obtained based on the prediction error (co)variances for single nucleotide polymorphisms (SNPs), which were obtained from the prediction error (co)variances of genomic predictions based on the inverse of the coefficient matrix and formulas to estimate SNP effects.

**Results:**

Computation of p-values took a negligible time for a dataset with almost 2 million animals in the pedigree and 1424 genotyped sires, and no inflation of statistics was observed. The SNPs that passed the Bonferroni threshold of 10^−5.9^ were the same as those that explained the highest proportion of additive genetic variance, but even at the same significance levels and effects, some of them explained less genetic variance due to lower allele frequency.

**Conclusions:**

The use of a p-value for SSGWAS is a very general and efficient strategy to identify quantitative trait loci (QTL). It can be used for complex datasets such as those used in animal breeding, where only a proportion of the pedigreed animals are genotyped.

## Background

With availability of high-density SNP genotypes, detection and mapping of causal genes and QTL in livestock genetics is usually accomplished by genome-wide association studies (GWAS). The most frequent GWAS method is single-marker fixed regression in a mixed linear model, in which genotypes at one marker are fit in the model as a covariate, and correction for the remaining genetic effects is based on a genetic relationship matrix. This is also known as efficient mixed-model association expedited (EMMAX) [1–3]. In human and livestock genetic studies, the associated variance components are often estimated once on the same dataset and then assumed as known, e.g. [4, 5] with negligible effects on the p-values computed [1]. However, the use of EMMAX requires all phenotyped individuals to be genotyped and vice versa. In livestock, this requirement is not met for, e.g. dairy bulls, that do not have sex-limited phenotypes (e.g. milk yield). In general, many animals that are phenotyped (e.g. for growth) would benefit from phenotypic information on relatives (e.g. growth in daughters, ancestors and collateral relatives). Typically, for GWAS using EMMAX, phenotypes of relatives are “projected” onto the genotyped animals [6–8] in a process called de-regression, which has been successfully used to detect and map QTL in livestock [9, 10].

De-regression is a cumbersome process and usually it is not optimal because it is an approximation that loses information and can lead to inaccuracies (e.g. spurious signals) due to selection not being accounted for, or by ignoring environmental effects. In particular, using de-regression leads to double-counting when the genotyped population includes both sires and their progeny. Legarra and Vitezica [11] proposed a more general two-trait variance component model for GWAS, where the two traits modeled are the phenotype and “gene content” (number of copies of the reference allele) at the marker. Single-marker EMMAX regression is a particular case of the model in [11] when all individuals are genotyped and phenotyped. However, the method in [11] would be very slow for a GWAS because it requires maximization of a restricted maximum likelihood (REML) at each marker.

The GBLUP or SNP-BLUP framework [12–14] allows for the joint estimation of marker effects and the automatic correction for genetic structure in the population. The so-called single-step methods [i.e. single-step GBLUP (SSGBLUP) and single-step SNP-BLUP (SSSNP-BLUP)] project genotypes onto phenotyped individuals, using pedigree relationships [15–18]. These “single-step methods” allow estimation of both breeding values and marker effects [17, 19, 20], and the latter have been used for GWAS analysis [21–23], typically based on the size of estimated marker effects or a related statistic such as the proportion of genetic variance explained by a marker or chromosome segment.

Proper GWAS analyses typically consider the estimates of either marker estimates or variance explained by segments, together with their uncertainty, to derive either p-values being different from 0 [1, 24, 25], or posterior probabilities of a region explaining more than a predetermined threshold [26, 27]. Both are proper statistical analyses with well-defined error rates. However, in most single-step methods used for GWAS (e.g. [21–23]), point estimates (posterior means) of marker effects are used, without accounting for their incertitude, because this is the output of common software. The use of “variances explained” is also often poorly implemented, because the variance explained has the form $2\sum p_{i} q_{i} \hat{a}_{i}^{2}$, which ignores the uncertainty on the estimate of marker effects *and* the linkage disequilibrium between markers. In other words, variances explained are reported as point estimates, without confidence intervals, p-values or posterior probabilities.

An additional source of confusion for the reader is the arbitrary choice of “windows” of adjacent SNPs, since, to date, there is no current consensus. As a result, studies are difficult to compare. For instance, [21] studied “the 20 largest explanatory loci”, [22] studied “the 10 windows explaining the largest amount of genomic variance for gene annotation, gene network and pathway analyses”, whereas [23] considered “1.5-Mb SNP windows that explained more than 0.50% of the genetic variance”. In these studies, there was no assessment (in the form of p-values or posterior probabilities) to check if the variances explained by these regions were not due to chance. Although using estimates of marker effects or explained variances considers correctly the *magnitude* of the estimated marker effects, it does not always consider correctly the *uncertainty* in the estimation of marker effects. Even worse, there is a possibility that different iterative schemes are used. All these problems render interpretation of signals more difficult and perhaps suboptimal [28].

Recently, equivalences between GBLUP and single-marker GWAS (EMMAX) results have been demonstrated in a series of papers [28–30]. In both cases, the statistic used is $\hat{a}_{i} /sd( {\hat{a}_{i} } )$ (i.e. the marker effect estimate over its standard deviation). Papers [28–30] proved that these statistics are mathematically equivalent, i.e. $\hat{a}_{i} /sd( {\hat{a}_{i} } )$ from EMMAX is equal to $\hat{a}_{i} /sd( {\hat{a}_{i} } )$ from GBLUP. This is remarkable because, in EMMAX, the effect of the marker is fitted as fixed and, in GBLUP, as random. An application and the comparison with Bayesian methods were performed on a dataset of gait in horses [25]. Still, the method using GBLUP results could not be applied to datasets that consisted of mixtures of genotyped and ungenotyped animals.

Lu et al. [31] showed that the same logic used in [28–30] for GBLUP can be used in a SSGBLUP context. Simply stated, to obtain a statistical test for the effect of a single marker, we need only estimates of the breeding values of genotyped animals and their sampling distribution, which can be readily obtained from SSGBLUP. Unfortunately, the article of Lu et al. [31] seems to have gone unnoticed because its main focus is on feed efficiency (not on methods for GWAS) and uses a small dataset (i.e. 7000 phenotyped animals of which 5000 are genotyped), and therefore the applicability to large datasets was unclear.

In the present work, we present the implementation of single-step GWAS (SSGWAS) with frequentist p-values, as in [31], together with an application on a very large beef cattle dataset. We describe algorithms and computational procedures along with their bottlenecks. We try to show that GWAS with frequentist p-values can be applied to quite large datasets, comparable to datasets used in many national evaluations. GWAS of the beef cattle dataset led to the detection of highly significant signals in marker loci that have already been described in the literature and showed good empirical behavior.

## Methods

### Theory

The classical GWAS method (EMMAX) for marker $i$ uses a linear model ${\mathbf{y}} = {\mathbf{Xb}} + {\mathbf{z}}_{i} a_{i} + {\mathbf{u}} + {\mathbf{e}}$, where vector ${\mathbf{b}}$ contains fixed effects, ${\mathbf{z}}_{i}$ is a vector with “gene contents” (0, 1, or 2 at the marker), $a_{i}$ is the allele substitution effect of the $i$th marker, and ${\mathbf{u}}$ is a vector of breeding values, modelled as ${\text{Var}}\left( {\mathbf{u}} \right) = \frac{{{\mathbf{ZZ^{\prime}}}}}{{2\sum p_{i} q_{i} }}\sigma_{u}^{2} = {\mathbf{G}}\sigma_{u}^{2}$, where ${\mathbf{G}}$ is a genomic relationship matrix and ${\mathbf{Z}}$ is a matrix with gene contents for all markers; $p_{i}$ $(q_{i}=1-p_{i})$ is the frequency of the reference allele at the $i$th marker. We assume, as customary [1], that variances $\sigma_{u}^{2}$ and $\sigma_{e}^{2}$ are assumed known, if needed from a single preliminary estimate from the same dataset. The normal hypothesis test for the effect of the marker uses the statistic $\frac{{\hat{a}_{i} }}{{sd( {\hat{a}_{i} } )}}$, where both values are obtained from the inversion of the coefficient matrix of the mixed model equations of the model. P-values testing whether the allele substitution effect differs from 0 are obtained as $pval_{i} = 2\left( {1 -\Phi \left( {\left| {\frac{{\hat{a}_{i} }}{{sd( {\hat{a}_{i} } )}}} \right|} \right)} \right)$, where $\Phi$ is the cumulative standard normal function.

It has been shown [28–30] that joint estimates of marker effects ${\mathbf{a}}$ from SNP-BLUP (or GBLUP models) of the form ${\mathbf{y}} = {\mathbf{Xb}} + {\mathbf{Za}} + {\mathbf{e}}$ with prior assumption ${\text{Var}}\left( {\mathbf{a}} \right) = {\mathbf{I}}\frac{{\sigma_{u}^{2} }}{{2\sum p_{i} q_{i} }}$ lead to the statistics $\frac{{\hat{a}_{i} }}{{sd\left( {a_{i} } \right)}}$ that are equivalent to the statistic obtained for marker $i$ in the EMMAX fixed regression framework.

The use of SSSNP-BLUP instead of SSGBLUP to obtain p-values is straightforward, because $\hat{a}_{i}$ and $sd( {\hat{a}_{i} } )$ are immediately available from the output of SSSNP-BLUP (e.g. [17]). In GBLUP and SSGBLUP, the same values can be obtained as linear transformations of the estimates of breeding values $\hat{u}_{i}$ and their prediction error (co)variances [25, 28–30]. These can be obtained from the inverse of the mixed model equations [32, 33].

### Algorithm

The algorithm for SSGWAS, which accommodates both genotyped and non-genotyped animals, has been implemented in the blupf90 suite of programs [34]. It combines the algorithms for SSGBLUP (e.g. [18]) and back-solving to obtain estimates of marker effects and their associated p-values from estimates of breeding values [30]:

1. Construct the inverse of the joint pedigree-genomic relationship matrix $${\mathbf{H}}^{ - 1} = {\mathbf{A}}^{ - 1} + \left( {\begin{array}{*{20}c} 0 & 0 \\ 0 & {{\mathbf{G}}^{ - 1} - {\mathbf{A}}_{22}^{ - 1} } \\ \end{array} } \right)$$, with $${\mathbf{H}} = \left( {\begin{array}{*{20}c} {{\mathbf{A}}_{11} - {\mathbf{A}}_{12} {\mathbf{A}}_{22}^{ - 1} {\mathbf{A}}_{21} + {\mathbf{A}}_{12} {\mathbf{A}}_{22}^{ - 1} {\mathbf{GA}}_{22}^{ - 1} {\mathbf{A}}_{21} } & {{\mathbf{A}}_{12} {\mathbf{A}}_{22}^{ - 1} {\mathbf{G}}} \\ {{\mathbf{GA}}_{22}^{ - 1} {\mathbf{A}}_{21} } & {\mathbf{G}} \\ \end{array} } \right)$$, which projects genomic relationships ${\mathbf{G}} = {\mathbf{ZZ^{\prime}}}/2\sum p_{i} q_{i}$ from genotyped animals (labelled as “2”) to non-genotyped animals (labelled as “1”). Matrix $${\mathbf{A}} = \left( {\begin{array}{*{20}c} {{\mathbf{A}}_{11} } & {{\mathbf{A}}_{12} } \\ {{\mathbf{A}}_{21} } & {{\mathbf{A}}_{22} } \\ \end{array} } \right)$$ is the pedigree-based relationship matrix. Matrix ${\mathbf{G}}$ is (usually) constructed as ${\mathbf{G}} = \left( {1 - \alpha } \right)\left( {a + b\frac{{{\mathbf{ZZ^{\prime}}}}}{{2\sum p_{i} q_{i} }}} \right) + \alpha {\mathbf{A}}_{22}$, where $a$ and $b$ are chosen to equate average inbreeding and average relationships in ${\mathbf{G}}$ and ${\mathbf{A}}_{22}$ and $\alpha$ is a small value (typically from 0 to 0.05). This results in genomic and pedigree relationships to be compatible [35, 36] and ${\mathbf{G}}$ is invertible [12]. Matrix ${\mathbf{Z}}$ contains centered gene content as in [12], but using observed allele frequencies. Other possibilities exist to create ${\mathbf{H}}^{ - 1}$ depending on model assumptions [37].

2. Construct the mixed model equations for SSGBLUP. In a simple case (a model with a single genetic effect) these would be:

$$\left( {\begin{array}{*{20}c} {{\mathbf{X^{\prime}X}}\upsigma_{\text{e}}^{ - 2} } & {{\mathbf{X^{\prime}W}}\upsigma_{\text{e}}^{ - 2} } \\ {{\mathbf{W^{\prime}X}}\upsigma_{\text{e}}^{ - 2} } & {{\mathbf{W^{\prime}W}}\upsigma_{\text{e}}^{ - 2} + {\mathbf{H}}^{ - 1}\upsigma_{\text{u}}^{ - 2} } \\ \end{array} } \right)\left( {\begin{array}{*{20}c} {{\hat{\varvec{\beta }}}} \\ {{\hat{\mathbf{u}}}} \\ \end{array} } \right) = \left( {\begin{array}{*{20}c} {{\mathbf{X^{\prime}y}}\upsigma_{\text{e}}^{ - 2} } \\ {{\mathbf{W^{\prime}y}}\upsigma_{\text{e}}^{ - 2} } \\ \end{array} } \right),$$

where ${\hat{\varvec{\beta }}}$ are estimates of fixed effects and ${\hat{\mathbf{u}}}$ are estimates of breeding values (not marker effects). More complex cases and multiple-trait models can be easily accommodated [31].

3. Factorize and obtain the sparse inverse of the coefficient matrix. The whole inverse cannot be obtained directly as it is typically too big [38]. Instead, a “sparse inverse” (${\mathbf{C}}$) is obtained with selected elements of the inverse that corresponds to the non-zero entries of a (sparse) Cholesky factor (${\mathbf{LL}} '$). In our case, this is achieved using supernodal sparse factorization and inversions as programmed in YAMS [39, 40]. Factorization is the computing bottleneck of the procedure and is roughly cubic on the number of genotyped animals; YAMS reduces the computing time by, roughly, one order of magnitude.

4. Solve the mixed model equations for $$\left( {\begin{array}{*{20}c} {{\hat{\varvec{\beta }}}} \\ {{\hat{\mathbf{u}}}} \\ \end{array} } \right)$$ by using the sparse Cholesky factor.

5. Extract from ${\mathbf{C}}$ the submatrix that corresponds to the genotyped animals, ${\mathbf{C}}^{{{\mathbf{u}}_{2} {\mathbf{u}}_{2} }}$, which contains the prediction error (co)variances of their estimated breeding values, ${\hat{\mathbf{u}}}_{2}$, i.e. ${\text{Var}}\left( {{\mathbf{u}} - {\hat{\mathbf{u}}}_{2} } \right)$.

6. Back-solve for SNP effect estimates using ${\hat{\mathbf{a}}}|{\hat{\mathbf{u}}} = \left( {1 - \alpha } \right)b{\mathbf{Z^{\prime}}}\frac{1}{{2\varSigma p_{i} q_{i} }}{\mathbf{G}}^{ - 1} {\hat{\mathbf{u}}}_{2}$.

If matrix ${\mathbf{G}}$ is full rank and compatible with pedigree relationships (for instance, if ${\mathbf{Z}}$ is built with base allele frequencies) then ${\hat{\mathbf{a}}}|{\hat{\mathbf{u}}} = {\mathbf{Z^{\prime}}}\frac{1}{{2\varSigma p_{i} q_{i} }}{\mathbf{G}}^{ - 1} {\hat{\mathbf{u}}}_{2}$ [12, 41, 42].

7. Obtain individual prediction error variances of SNP effect estimates as [30]:

$${\text{Var}}\left( {\hat{a}_{i} } \right) = \frac{1}{{2\sum p_{i} q_{i} }}\left( {1 - \alpha } \right)b{\mathbf{z}}_{\text{i}}^{'} {\mathbf{G}}^{ - 1} \left( {{\mathbf{G}}\upsigma_{\text{u}}^{2} - {\mathbf{C}}^{{{\mathbf{u}}_{2} {\mathbf{u}}_{2} }} } \right){\mathbf{G}}^{ - 1} {\mathbf{z}}_{{\mathbf{i}}} \left( {1 - \alpha } \right)b\frac{1}{{2\sum p_{i} q_{i} }},$$

where ${\mathbf{z}}_{{\mathbf{i}}}$ is the $i$-th column of ${\mathbf{Z}}$, corresponding to genotypes of marker $i$ across individuals. The values of $\alpha$ in steps 6 and 7 refer to the “blending” of ${\mathbf{G}}$ with ${\mathbf{A}}$ in step 1 and will change with different choices of blending parameters.

8. The p-value for marker $i$ is obtained as $pval_{i} = 2\left( {1 -\Phi \left( {\left| {\frac{{\hat{a}_{i} }}{{sd( {\hat{a}_{i} } )}}} \right|} \right)} \right)$.

Note that this analysis has to be run only once, as opposed to the $n$ individual runs for $n$ markers in the classical “fixed effect” regression or EMMAX (e.g. [43]). Note that within the framework of SSSNP-BLUP, the estimates of SNP effects and their variance are obtained directly without steps 1 to 6. From expressions in [29], it is possible to convert estimates of the random marker effects, $\hat{a}_{i}$, to the fixed regression estimates, $\hat{b}_{i}$, using $\hat{b}_{i} = \frac{{Var\left( {\hat{b}_{i} } \right)}}{{\sigma_{u}^{2} }}\hat{a}_{i}$, with $Var\left( {\hat{b}_{i} } \right) = \frac{{\left( {\sigma_{u}^{2} } \right)^{2} }}{{Var( {\hat{a}_{i} } )}}$, which results in $\hat{b}_{i} = \frac{{\sigma_{u}^{2} }}{{Var( {\hat{a}_{i} } )}}\hat{a}_{i} .$

### Data

We re-analyzed a dataset on birth weight from the American Angus Association [44]. The complete dataset is very large, with about 7 million individual weights, 52,000 genotyped animals and 8 million animals in the pedigree, and the SSGBLUP evaluations cannot be run in core. Thus, we used only phenotypes recorded in the last 4 years, specifically from 2009 to 2012, which comprised 1,046,623 birth weights. Three generations of ancestors were traced back, totaling 1,849,865 individuals. All available genotypes of sires with phenotyped offspring were used (i.e. 1424 genotyped sires). Other genotyped animals were not considered since they include selection candidates (with no phenotypic information) and elite cows, which do not represent the population well. Genotypes were obtained with the BovineSNP50k v2 BeadChip; 38,122 polymorphic SNPs were used after quality control [44].

The linear model for birth weight included the effects of contemporary group, animal breeding values and the permanent environmental effect of the mother, which considers maternal ability during pregnancy. This differs from the model actually used in national evaluations, which also considers a maternal genetic effect. Variance components were fixed at values used in the national evaluation, with a heritability of 0.48 and a maternal component of 0.10.

Computations were done using the blupf90 software suite and GWAS results were plotted with qqman [45]. Rejection thresholds used a Bonferroni correction for multiple testing of 0.05/38,122, which equals 5.9 on the $- \log 10$ scale. Significant regions were explored in AnimalQTLdb and Jbrowse [46] using the bovine map assembly UMD 3.1 [47]. Although a new genome map assembly has already been published (i.e. ARS-UCD 1.2), the aforementioned genome browsers still use the UMD 3.1 assembly.

In addition to SSGWAS p-values, we plotted GWAS results based on the percentage of variance explained by marker effects [20]. This estimates the population genetic variance explained by the marker effect, and is approximately computed as $2p_{i} q_{i} \hat{a}_{i}^{2}$. There are no theoretical thresholds in this approach, and we used an arbitrary threshold of 0.10% of total genetic variance explained by one marker. Note that there is no formal assessment of this hypothesis: neither the p-values nor the posterior distributions are obtained for the “variance explained”. Opposite to [20], we do not present results of iterating the SSGBLUP using “weights” for each marker, as this procedure did not result in increased marker effect and variance (results not shown).

## Results

**Fig. 1:**
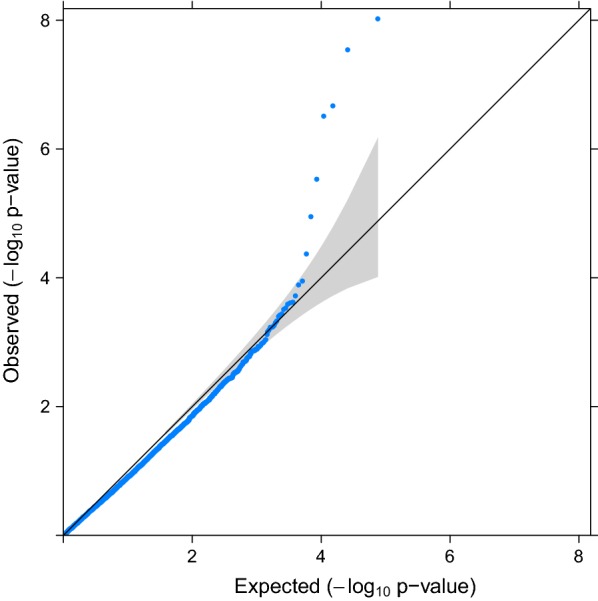
Quantile-quantile plot (QQPLOT) for the $- \log 10\left( {pvalue} \right).$ The grey region represents a 95% confidence interval

**Fig. 2:**
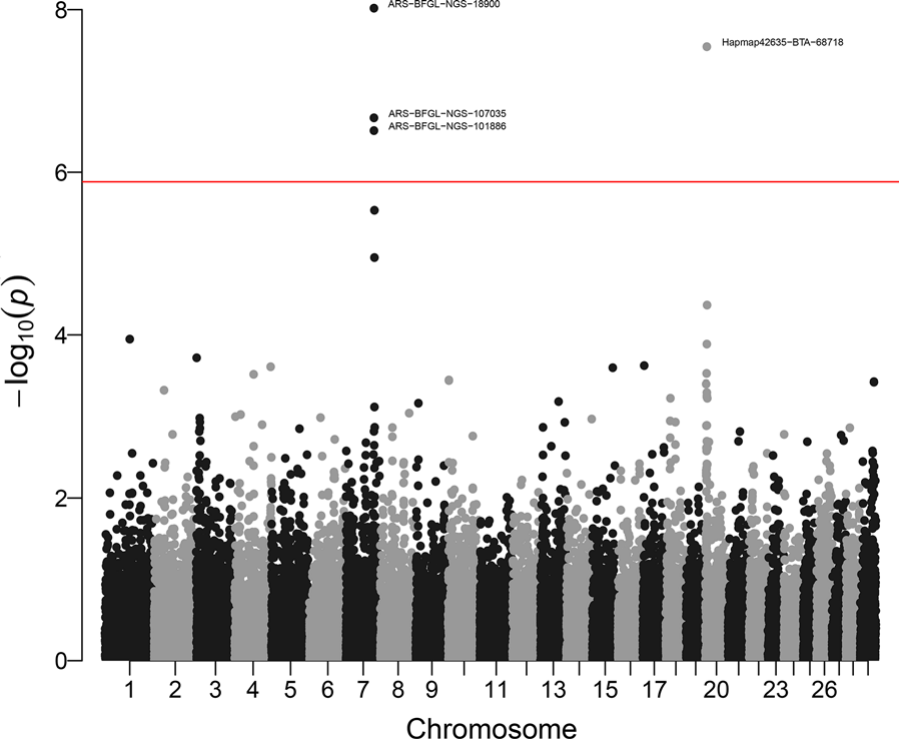
Manhattan plot (p-values of individual SNP effects) for birth weight. The red line corresponds to the Bonferroni rejection threshold for nominal alpha = 0.05

Factorization of the mixed model equations and extraction of ${\mathbf{C}}^{{{\mathbf{u}}_{2} {\mathbf{u}}_{2} }}$ required 30 Gb of RAM memory and 14 h (wall-clock time). Computation of $Var( {\hat{a}_{i} } )$ and p-values took only a few minutes. Quantile–quantile plot and Manhattan plots are in Figs. 1 and 2. The quantile–quantile plot did not show large deviations from the null hypothesis, which means that SSGBLUP correctly captured the structure of the population through the relationship matrices. When population structure is not accounted for, inflation of GWAS signals (in our case, $\hat{a}_{i} /sd( {\hat{a}_{i} } )$ or $- \log 10\left( {pvalue} \right)$) is expected [3, 48, 49].

The GWAS results pointed to two chromosome regions that are significant at the genome-wide level, with values of $- \log 10\left( {pvalue} \right)$ near 8. The region at the end of chromosome 7 includes three markers: ARS-BFGL-NGS-107035, ARS-BFGL-NGS-101886 and ARS-BFGL-NGS-18900, which were in very high linkage disequilibrium with each other (correlation between genotypes > 0.95 in all cases). *AnimalQTLdb* reports signals in the same region for “body conformation” in Holstein cattle [10] and for “average daily body weight gain” in Brangus cattle (composite of Brahman and Angus) [50], which indicates that our finding is not a false positive. The second region at the end of chromosome 20 only includes marker Hapmap42635-BTA-68718, which is included in a QTL region for mid-test body weight that was detected in the Hereford breed [51].

**Fig. 3:**
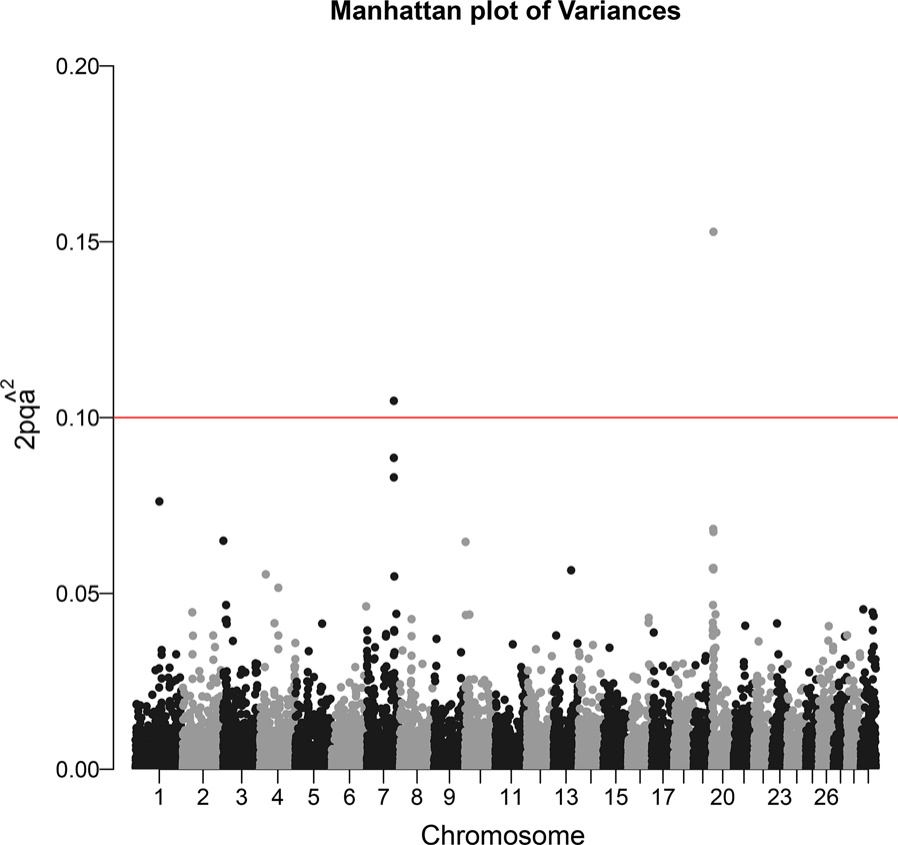
Percentage of genetic variance explained by markers for birth weight in American Angus. The red line corresponds to an arbitrary rejection threshold of 0.10%

Figure 3 shows estimates of the percentage of genetic variance attributed to each marker (as $2p_{i} q_{i} \hat{a}_{i}^{2}$) based on estimates of marker effects from SSGBLUP. The top markers are the same as in Fig. 2 (ARS-BFGL-NGS-18900 and Hapmap42635-BTA-68718), but the region on chromosome 7 has a smaller peak than the region on chromosome 20. At first sight, this raises questions, because estimates of marker effects ± standard errors were similar for the peaks on chromosomes 7 and 20 (− 0.041 ± 0.007 and 0.043 ± 0.008, respectively). The different relative heights of the peaks on chromosomes 7 and 20 in the variance plot (Fig. 3) and in the $- \log 10\left( {pvalue} \right)$ plot (Fig. 2) are entirely due to the different minor allelic frequencies: 0.28 and 0.46, respectively, which enter into the estimator of explained variance $2p_{i} q_{i} \hat{a}_{i}^{2}$. For the marker on chromosome 7, the variance explained is $2 \times 0.28 \times \left( {1 - 0.28} \right) \times \left( { - 0.041} \right)^{2} = 0.000678$, whereas for the marker on chromosome 20, it is $2 \times 0.46 \times \left( {1 - 0.46} \right) \times 0.043^{2} = 0.000919$. In the case of these two markers, the estimates of the fixed marker effects are also nearly identical (not shown). In other words, these two figures explain two different things, i.e. Figure 2 explains whether markers have apparent effects that are seemingly different from 0 (with statistical assessment) and Fig. 3 explains whether markers explain part of the genetic variance—but with no statistical assessment.

## Discussion

Single-step methods can include genotyped and ungenotyped animals in a single genomic analysis. Estimates of SNP effects from single-step methods became available in 2012 [20, 52]. However, to date, a measure of the significance for SNP estimates has not been available in current implementations of single-step GBLUP. Expanding the ideas based on the equivalence between GBLUP and single-marker GWAS [28–30], Lu et al. [31] derived GWAS within a ssGBLUP framework for a relatively small dataset. In our study, we show, for the first time, the acquisition of p-values calculated by using a large dataset including genotypes, phenotypes, and pedigree.

In this paper, we addressed frequentist statistics for GWAS. Single-step Bayesian methods using mixtures of distributions exist [17], and the posterior probabilities that they report may better control the error rate in discoveries; we refer the reader to Fernando et al. [26] for a thorough review and discussion. Here, we discuss models that assume multivariate normality, i.e. for which posterior probabilities are not explicitly obtained. However, Bayes factors can be easily obtained from $\hat{a}_{i}$ and $var( {\hat{a}_{i} } )$ [25], and they can be transformed into posterior probabilities.

Known associations from studies on beef cattle in the literature show good empirical agreement with our GWAS findings. The quantile–quantile plot showed no inflation of p-values, as expected, because the structure of relationships was well accounted for [2, 3, 48]. Compared to EMMAX, SSGWAS is a more comprehensive method that can include phenotypes from non-genotyped individuals. Additional advantages compared to “approximate” SSGWAS include avoiding the need for arbitrary choices, such as the length of segments or the use of iterative schemes. The use of the percentage of explained variance, as advocated by several authors [20, 27], needs to be done with formal testing, either frequentist or Bayesian. Explained variance is useful for breeding purposes, but may not be useful for QTL detection if the final objective is to have a detailed understanding of the action of genes. In particular, point estimates of explained variance using $2\sum p_{i} q_{i} \hat{a}_{i}^{2}$ are neither statistics with defined distributions nor posterior probabilities.

The exact form of matrix ${\mathbf{G}}$ depends on modelling assumptions. The weight $\left( {1 - \alpha } \right)$ given to markers in ${\mathbf{G}} = \left( {1 - \alpha } \right)\left( {a + b\frac{{{\mathbf{ZZ^{\prime}}}}}{{2\sum p_{i} q_{i} }}} \right) + \alpha {\mathbf{A}}_{22}$ depends on the proportion of additive genetic variance that is explained by the markers, which can be estimated by REML [16]. Our experience shows that GEBV are fairly insensitive to values of $\left( {1 - \alpha } \right)$ between 0.95 and 1, and estimates of SNP effects should not change much. The values of $a$ and $b$ depend (basically) on the structure of the genotypes of the population, but they can be obtained from the data.

Although SSGWAS could be implemented for a dataset that included the last 4 years of phenotypes and all genotyped bulls present in the American Angus data, it was computationally not feasible with the entire dataset. Even for the reduced dataset, the computational burden for the factorization of the left-hand side of the mixed model equations was not negligible. For this dataset, ${\mathbf{H}}^{ - 1}$ contained (roughly) 16 million non-null elements from pedigree data and 2 million non-null elements from genotypes. However, ${\mathbf{H}}^{ - 1}$ for the whole dataset in Lourenco et al. [44] contained 63 million non-null elements from pedigree data and 2704 million non-null elements from genotypes. One simple method to reduce the size of the problem is to include only genotyped ancestors in SSGWAS and exclude genotyped selection candidates. This leads to a number of genotyped animals in the thousands or tens of thousands. Another strategy is to use a sparse version of ${\mathbf{G}}^{ - 1}$ based on the APY (algorithm for proven and young) approach [44, 53, 54], which substantially increases the number of null elements in ${\mathbf{G}}^{ - 1}$. If animals in the APY core portion of ${\mathbf{G}}$ are a representative sample of the population, this also improves estimates of the SNP effects [55]. However, the use of the APY approach for SSGWAS on marker effect estimates and p-values should be explored further. Overall, our method is appropriate for data in the order of several thousands of genotyped individuals and several millions of phenotypes and non-genotyped individuals. This includes the datasets used in many national and private company genetic evaluations but it does not include the very large evaluations, such as used in dairy cattle.

We emphasize that, contrary to regular single-marker GWAS, SSGWAS do not require computations to be repeated at each marker. Instead, all p-values are obtained in a single run of SSGWAS.

## Conclusions

Single-step GWAS is a very general and efficient strategy for the detection, localization and testing of QTL, providing frequentist p-values of marker effects. It can be used in complex datasets such as those used in animal breeding, with many unbalanced effects, very complex mixed linear models and the presence of genotyped and ungenotyped animals. Our proposed strategy is computationally viable for very large populations and solves the main issues in single-step GWAS that precluded use of the method.

## Data Availability

The data that support the findings of this study were provided from the American Angus Association but restrictions apply to the availability of these data, which were used under license for the current study, and thus are not publicly available. The methods described here are included using “OPTION snp_p_value” in the parameter file in software blupf90 (factorization of the mixed model equations and solving of the SSGBLUP equations) and postGSf90 (backsolving of snp effects and computation of p-values), available at http://nce.ads.uga.edu/software/.

## References

[1] Kang HM, Sul JH, Service SK, Zaitlen NA, Kong S, Freimer NB, Sabatti C (2010). Variance component model to account for sample structure in genome-wide association studies. Nat Genet.

[2] Kennedy BW, Quinton M, Van Arendonk JA (1992). Estimation of effects of single genes on quantitative traits. J Anim Sci.

[3] Teyssèdre S, Elsen JM, Ricard A (2012). Statistical distributions of test statistics used for quantitative trait association mapping in structured populations. Genet Sel Evol.

[4] Sahana G, Guldbrandtsen B, Thomsen B, Holm LE, Panitz F, Brøndum RF (2014). Genome-wide association study using high-density single nucleotide polymorphism arrays and whole-genome sequences for clinical mastitis traits in dairy cattle. J Dairy Sci.

[5] Hayes BJ, Pryce J, Chamberlain AJ, Bowman PJ, Goddard ME (2010). Genetic architecture of complex traits and accuracy of genomic prediction: coat colour, milk-fat percentage, and type in Holstein cattle as contrasting model traits. PLoS Genet.

[6] VanRaden PM, Wiggans GR (1991). Derivation, calculation, and use of national animal model information. J Dairy Sci.

[7] Garrick DJ, Taylor JF, Fernando RL (2009). Deregressing estimated breeding values and weighting information for genomic regression analyses. Genet Sel Evol..

[8] Ricard A, Danvy S, Legarra A (2013). Computation of deregressed proofs for genomic selection when own phenotypes exist with an application in French show-jumping horses. J Anim Sci.

[9] Rupp R, Senin P, Sarry J, Allain C, Tasca C, Ligat L (2015). A point mutation in suppressor of cytokine signalling 2 (*Socs2*) increases the susceptibility to inflammation of the mammary gland while associated with higher body weight and size and higher milk production in a sheep model. PLoS Genet.

[10] Cole JB, Wiggans GR, Ma L, Sonstegard TS, Lawlor TJ, Crooker BA (2011). Genome-wide association analysis of thirty one production, health, reproduction and body conformation traits in contemporary U.S. Holstein cows. BMC Genomics.

[11] Legarra A, Vitezica ZG (2015). Genetic evaluation with major genes and polygenic inheritance when some animals are not genotyped using gene content multiple-trait BLUP. Genet Sel Evol..

[12] VanRaden PM (2008). Efficient methods to compute genomic predictions. J Dairy Sci.

[13] Yang J, Benyamin B, McEvoy BP, Gordon S, Henders AK, Nyholt DR (2010). Common SNPs explain a large proportion of the heritability for human height. Nat Genet.

[14] Fernando RL, Habier D, Stricker C, Dekkers JCM, Totir LR (2007). Genomic selection. Acta Agric Scand A Anim Sci..

[15] Aguilar I, Misztal I, Johnson D, Legarra A, Tsuruta S, Lawlor T (2010). Hot topic: a unified approach to utilize phenotypic, full pedigree, and genomic information for genetic evaluation of Holstein final score. J Dairy Sci.

[16] Christensen OF, Lund MS (2010). Genomic prediction when some animals are not genotyped. Genet Sel Evol..

[17] Fernando RL, Dekkers JC, Garrick DJ (2014). A class of Bayesian methods to combine large numbers of genotyped and non-genotyped animals for whole-genome analyses. Genet Sel Evol..

[18] Legarra A, Christensen OF, Aguilar I, Misztal I (2014). Single step, a general approach for genomic selection. Livest Sci..

[19] Legarra A, Ducrocq V (2012). Computational strategies for national integration of phenotypic, genomic, and pedigree data in a single-step best linear unbiased prediction. J Dairy Sci.

[20] Wang H, Misztal I, Aguilar I, Legarra A, Muir WM (2012). Genome-wide association mapping including phenotypes from relatives without genotypes. Genet Res (Camb)..

[21] Dikmen S, Cole JB, Null DJ, Hansen PJ (2013). Genome-wide association mapping for identification of quantitative trait loci for rectal temperature during heat stress in Holstein cattle. PLoS One.

[22] Tiezzi F, Parker-Gaddis KL, Cole JB, Clay JS, Maltecca C (2015). A genome-wide association study for clinical mastitis in first parity US Holstein cows using single-step approach and genomic matrix re-weighting procedure. PLoS One.

[23] Han Y, Peñagaricano F (2016). Unravelling the genomic architecture of bull fertility in Holstein cattle. BMC Genet.

[24] Nagamine Y, Pong-Wong R, Navarro P, Vitart V, Hayward C, Rudan I (2012). Localising loci underlying complex trait variation using regional genomic relationship mapping. PLoS One.

[25] Legarra A, Ricard A, Varona L (2018). GWAS by GBLUP: single and multimarker EMMAX and Bayes factors, with an example in detection of a major gene for horse gait. G3 (Bethesda).

[26] Fernando R, Toosi A, Wolc A, Garrick D, Dekkers J (2017). Application of whole-genome prediction methods for genome-wide association studies: a Bayesian approach. J Agric Biol Environ Stat..

[27] Fernando Rohan L., Garrick Dorian (2013). Bayesian Methods Applied to GWAS. Methods in Molecular Biology.

[28] Chen C, Steibel JP, Tempelman RJ (2017). Genome-wide association analyses based on broadly different specifications for prior distributions, genomic windows, and estimation methods. Genetics.

[29] Bernal Rubio YL, Gualdrón Duarte JL, Bates RO, Ernst CW, Nonneman D, Rohrer GA (2016). Meta-analysis of genome-wide association from genomic prediction models. Anim Genet.

[30] Gualdrón Duarte JL, Cantet RJ, Bates RO, Ernst CW, Raney NE, Steibel JP (2014). Rapid screening for phenotype-genotype associations by linear transformations of genomic evaluations. BMC Bioinformatics.

[31] Lu Y, Vandehaar MJ, Spurlock DM, Weigel KA, Armentano LE, Connor EE (2018). Genome-wide association analyses based on a multiple-trait approach for modeling feed efficiency. J Dairy Sci.

[32] Henderson CR (1975). Best linear unbiased estimation and prediction under a selection model. Biometrics.

[33] Henderson CR (1984). Applications of linear models in animal breeding.

[34] Aguilar I, Tsuruta S, Masuda Y, Lourenco D, Legarra A, Misztal I. BLUPF90 suite of programs for animal breeding with focus on genomics. In: Proceedings of the world congress on genetics applied to livestock production: 11–16 February 2018; Auckland 2018.

[35] Vitezica ZG, Aguilar I, Misztal I, Legarra A (2011). Bias in genomic predictions for populations under selection. Genet Res (Camb)..

[36] Christensen OF, Madsen P, Nielsen B, Ostersen T, Su G (2012). Single-step methods for genomic evaluation in pigs. Animal.

[37] Christensen OF (2012). Compatibility of pedigree-based and marker-based relationship matrices for single-step genetic evaluation. Genet Sel Evol..

[38] Misztal I, Perez-Enciso M (1993). Sparse matrix inversion for restricted maximum likelihood estimation of variance components by expectation-maximization. J Dairy Sci.

[39] Masuda Y, Baba T, Suzuki M (2014). Application of supernodal sparse factorization and inversion to the estimation of (co) variance components by residual maximum likelihood. J Anim Breed Genet.

[40] Masuda Y, Aguilar I, Tsuruta S, Misztal I (2015). Technical note: acceleration of sparse operations for average-information REML analyses with supernodal methods and sparse-storage refinements. J Anim Sci.

[41] Strandén I, Garrick DJ (2009). Technical note: derivation of equivalent computing algorithms for genomic predictions and reliabilities of animal merit. J Dairy Sci.

[42] Misztal I, Wang H, Aguilar I, Legarra A, Muir B. Genome-wide association mapping using single-step GBLUP. In: Proceedings of the 63rd annual meeting of the european association of animal production: 27–31 August 2012; Bratislava; 2012.

[43] Zhou X, Stephens M (2012). Genome-wide efficient mixed-model analysis for association studies. Nat Genet.

[44] Lourenco D, Tsuruta S, Fragomeni BO, Masuda Y, Aguilar I, Legarra A (2015). Genetic evaluation using single-step genomic best linear unbiased predictor in American Angus. J Anim Sci.

[45] Turner SD (2018). qqman: an R package for visualizing GWAS results using Q–Q and manhattan plots. J Open Source Softw.

[46] Hu Z-L, Fritz ER, Reecy JM (2007). AnimalQTLdb: a livestock QTL database tool set for positional QTL information mining and beyond. Nucleic Acids Res.

[47] Zimin AV, Delcher AL, Florea L, Kelley DR, Schatz MC, Puiu D (2009). A whole-genome assembly of the domestic cow, *Bos taurus*. Genome Biol..

[48] Yang J, Zaitlen NA, Goddard ME, Visscher PM, Price AL (2014). Advantages and pitfalls in the application of mixed model association methods. Nat Genet.

[49] Devlin B, Roeder K (1999). Genomic control for association studies. Biometrics.

[50] Peters SO, Kizilkaya K, Garrick DJ, Fernando RL, Reecy JM, Weaber RL (2012). Bayesian genome-wide association analysis of growth and yearling ultrasound measures of carcass traits in Brangus heifers. J Anim Sci.

[51] Saatchi M, Beever JE, Decker JE, Faulkner DB, Freetly HC, Hansen SL (2014). QTLs associated with dry matter intake, metabolic mid-test weight, growth and feed efficiency have little overlap across 4 beef cattle studies. BMC Genomics.

[52] Wang H, Misztal I, Aguilar I, Legarra A, Fernando RL, Vitezica Z (2014). Genome-wide association mapping including phenotypes from relatives without genotypes in a single-step (ssGWAS) for 6-week body weight in broiler chickens. Front Genet..

[53] Misztal I (2016). Inexpensive computation of the inverse of the genomic relationship matrix in populations with small effective population size. Genetics.

[54] Masuda Y, Misztal I, Tsuruta S, Lourenco DA, Fragomeni BO, Legarra A (2015). Single-step genomic evaluations with 570 K genotyped animals in US Holsteins. Interbull Bull..

[55] Lourenco DAL, Legarra A, Moser D, Miller S, Misztal I (2018). Tuning indirect predictions based on SNP effects from single-step GBLUP. Interbull Bull..

